# Person-centered diagnosis and treatment in mental health. A model for empowering clients

**Published:** 2012-12-18

**Authors:** Yves Couturier

**Affiliations:** University of Sherbrooke, Canada E-mail: Yves.Couturier@USherbrooke.ca

## A book proposing a collaborative approach between practitioners and clients

For a long time, the mental health field helped to enhance innovation regarding services organization. It began with a strong desire to humanize patient care for persons having mental health disorders by supporting professional practices leading to the empowerment of clients. Adopting this perspective, Ladd and Churchill proposes in their book the utilization of a clinico-pragmatical model of co-construction (in which the practitioner and the client work together) of diagnosis and plan of treatment for clients suffering from mental health disorders.

The book is divided into three sections. The first part, which is in fact the opening chapter of the book, explains succinctly the axiological stance adopted by the two authors and formulates clearly the narrative strategy underlying the 15 following chapters. The second part contains eight chapters (Chapter 2 to Chapter 9), each of the chapters addressing one specific mental health disorder (for example, borderline personality disorder, compulsive personality, post-traumatic stress syndrome, etc.). The third section regroups seven chapters (Chapter 10 to Chapter 16), presenting respectively one particular mental health pattern (for example, bullying, self-hatred, compassion fatigue, etc.).

The authors suggest the utilization of a clinico-pragmatical approach, instead of using an etiological and scientific approach, to make a diagnosis. While the Diagnostic and Statistical Manual of Mental Disorders (DSM-IV) enumerates a list of symptoms that the diagnosing practitioner will try to identify in a patient, Ladd and Churchill propose to analyze clinical situations with the aid of a pragmatic ‘model’, which describes in general the various behaviours exhibited by a majority of persons suffering from a given mental disorder. For example, in the case of a person having an attention-deficit hyperactive disorder, agitation provokes a loss of focus that leads to control issues, which produces humiliation and frustration, which in turn cause difficult transitions, and so on. In sum, this model is essentially based on the observation of behaviors that individuals have in their everyday life, which is, by the way, very useful for the case manager. This pragmatic dimension characterizing the model helps the client to understand what is affecting him, and, consequently, to analyse himself his mental health disorder to his actual situation. According to the authors, the focalisation on the lived experience of clients, instead of relying on the classical medical etiology, establishes a new form of treatment allowing for the empowerment of the client.

All 15 thematical chapters are structured exactly in the same manner. They begin with the description of a clinico-pragmatical model for each mental health disorder and each mental health pattern that are described in the book. For each model, six specific behaviours are described. These behaviours, explained through the presentation of a clinical case and accompanied with collaborative diagnosis and treatment, are linked to each other in a circular manner. Finally, the authors give a number of very general instruments that can help to support the work of the practitioner, who will try to adapt each model to the particular clinical situation in which he is committed.

## A double distanciation from science

The book distances itself in two ways from science’s vital contribution to the diagnosis and treatment of mental health disorders. Building on the anti-psychiatry tradition, the book rejects the entrance in the health care system using the etiological argument, which is embodied in an archetypal manner in the DSM-IV. This classical criticism is more the object of an axiological postulate that the outcome of a thorough and comprehensive analysis of real professional practices that is supported by the utilization of this instrument. However, it seems fair to recognize that this criticism was historically fruitful in allowing for the renewal of the practice in the mental health field. The 15 clinico-pragmatical models proposed by the Ladd and Churchill could have been based on empirical scientific works, which are however numerous in the field in question, that do not adhere to the sole etiological rationale. Now, the book quotes very few scientific researches that could substantiate the point of view of the authors regarding criteria they decided to retain in order to construct their models.

This distanciation from science is also observable in the lack of reference to actual researches pertaining to key concepts such as person-centered care and empowerment, which are not clearly defined in the text. And yet, for these specific topics, conceptual advances are numerous.

## A multiple distanciation from reality

Despite Ladd and Churchill’s claim that they adhere to a pragmatical perspective, many aspects of the reality are not covered in the book. For example, the authors do not take into consideration the different national legislative frameworks, which, in many cases, forbid clinicians who are not physicians to make a diagnosis. In the same vein, a number of topics (the relatives of persons having mental health disorders, the environment in which these persons are living, the organization of services to which they have access, comorbidity, etc.) were not discussed in the book.

Yet, the conceptual proposal in their book would easily help in conceiving the notion of comorbidity by articulating many models. In addition, the authors suggest, in their editorial choice, that 50 years of anti-psychiatry do not produce any effect at all. But, even if it is undoubtedly a difficult task, we note that diagnosis and plans of treatment are already made according to a collaborative approach currently, a practice corresponding to the general desire to humanize these practices. One has the impression that the book is written by practitioners belonging to the fringe of partisans of evidence-based medicine. Yet, in fact, the services for persons suffering from mental health disorders are for the most part provided by social workers, nurses, and other professionals working for non-governmental organizations.

## Contribution of the book to the services integration

Explicitly, the book does not address at all the problem of service integration, Ladd and Churchill restricting their brief reflection on the issue to an argument that is commonplace, that is to say, service integration acts as an agent of practice standardization reaching the project of empowerment of clients. Does this mean that the well-learned readership of *International Journal of Integrated Care* has to put this book aside? We do not think so, for two reasons.

Firstly, any recollection of the project relating to empowerment of the patients is useful for the practice of case management. In this regard, Ladd and Churchill suggest an efficient manner to achieve that purpose. The book helps to develop a more fundamental and more durable therapeutic alliance between the practitioner and the client by giving the latter the capacity to make its own contribution in the definition of his mental health problem.

Then, beyond the official diagnosis, the case manager, which is by nature a generalist on these subjects, can utilize in a very relevant manner the different clinico-pragmatical models proposed by the authors in order to give meaning to the clinical situation in which he is responsible for service coordination.

Is spite of their low level of scientific quality, these models will allow the case manager to understand, modulate and adapt, to a certain extent of course, a diagnosis made by an expert in the context of the integrated service plan. In that sense, the clinico-pragmatical models elaborated by Ladd and Churchill could allow persons who are not experts in the domain of psychiatry to develop and utilize an interdisciplinary vocabulary focusing on the functional level of a mental health disorder, which will always be experienced more largely than in its sole psychiatric dimension. Yet, this widening from the clinical field to the sphere of experience, in particular regarding what can be done in service organization, is at the very heart of the work of any services coordinator.

